# Supplementary material for: Moving from drought hazard to impact forecasts
Source: Nat Commun. 2019 Oct 30;10:4945. doi: 10.1038/s41467-019-12840-z (PMC6821769; doi:10.1038/s41467-019-12840-z)
Supplement: Supplementary file 1 — Supplementary Information [file 41467_2019_12840_MOESM1_ESM.pdf]

Supplementary information

Moving from drought hazard to impact forecasts

Sutanto et al.

The Supplementary information comprises five Supplementary Figures, one Supplementary Notes, and Supplementary References.

Supplementary Figures

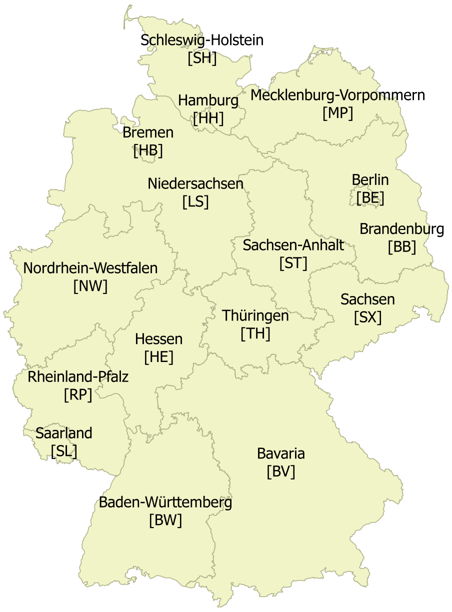

Supplementary Figure 1. Germany divided into NUTS-1 regions, including their acronyms.

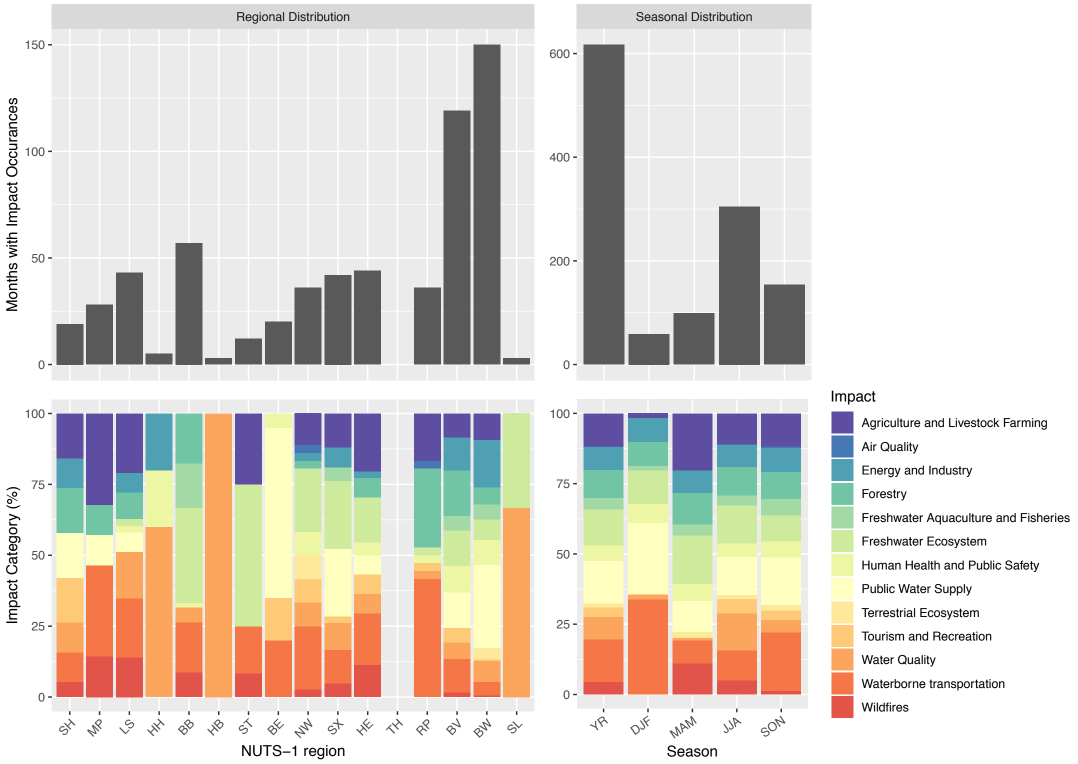

Supplementary Figure 2. Number of impact occurrences (top), and distribution of impact categories for each NUTS-1 region in Germany (lower left) and for each season (lower right) within the time frame of 1990-2017 obtained from the EDII[1]. For the acronym of NUTS-1 regions in Germany, see supplementary Fig. 1.

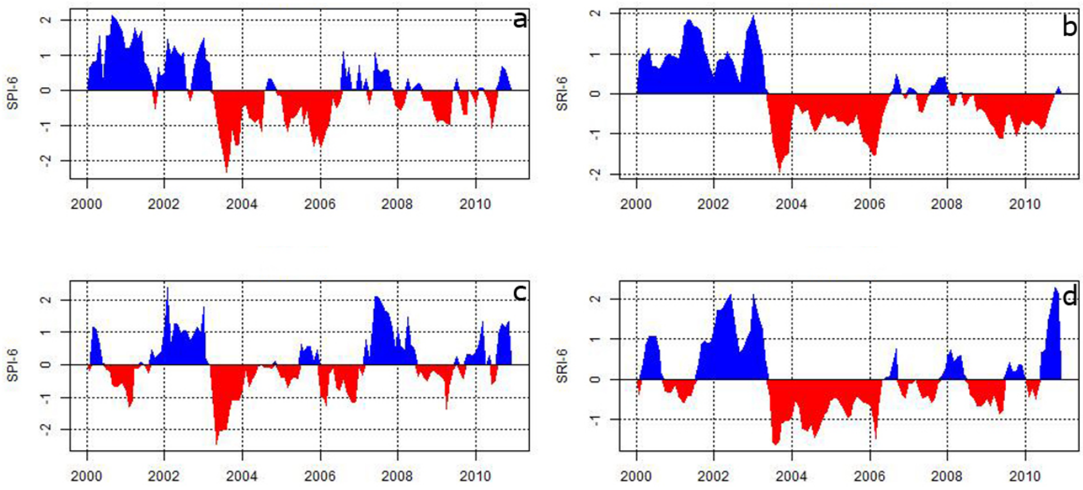

Supplementary Figure 3. Standardized Precipitation Index and Standardized Runoff Index accumulated over 6 months: (a) SPI-6 for Rheinland-Pfalz (RP); (b) SRI-6 for Rheinland-Pfalz (RP); (c) SPI-6 for Brandenburg (BB); (d) SRI-6 for Brandenburg (BB). SPI-6 and SRI-6 were calculated using proxy observed weather data.

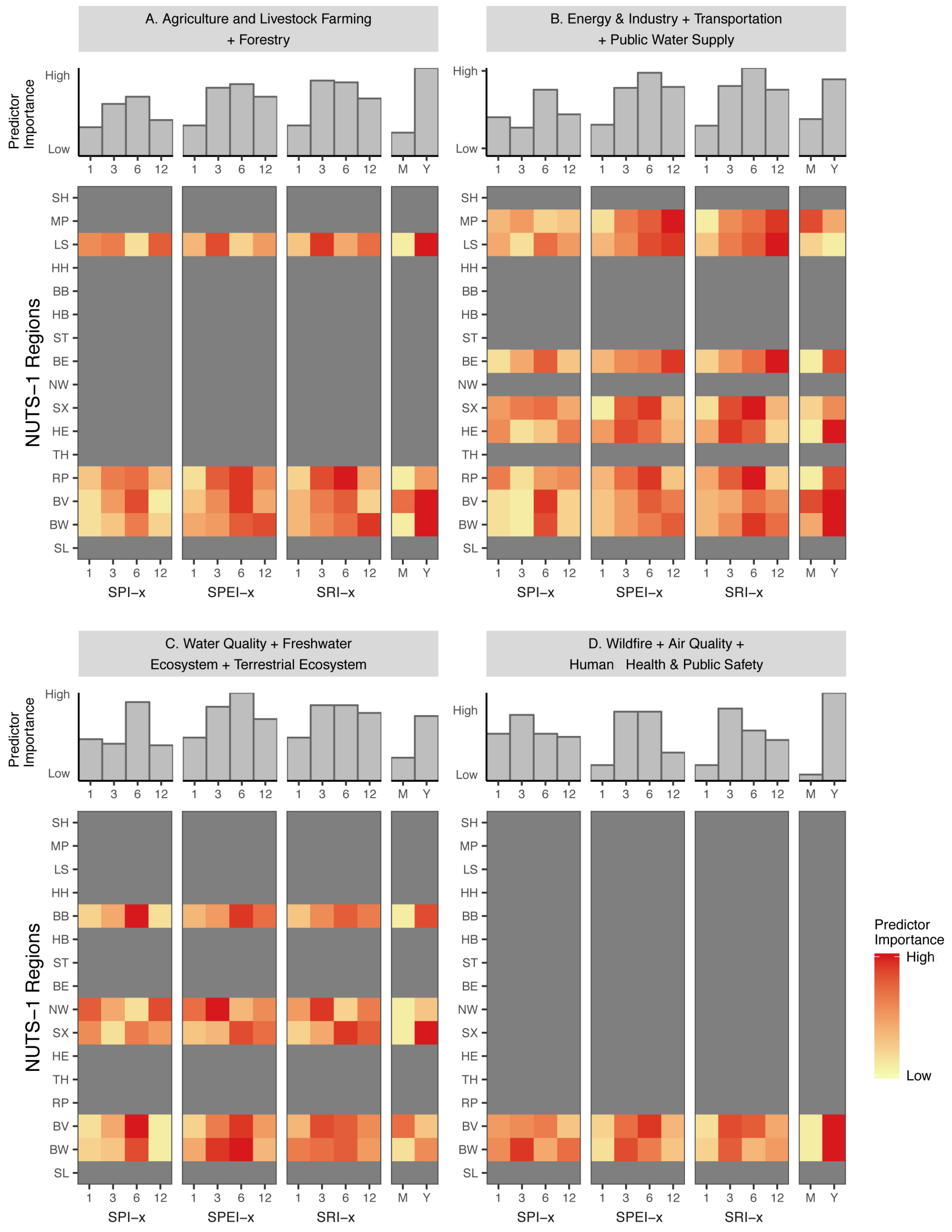

Supplementary Figure 4. Predictor importance in developed drought impact forecasting functions using Random Forest for impact Group 1 (a), Group 2 (b), Group 3 (c), and Group 4 (d). The colored boxes show the predictor importance for each NUTS-1 region in Germany. Red colors indicate highly-related predictors and yellow colors indicate lowly-related predictors to the selected drought impacts. A summary is given as histograms at top of each figure. Gray boxes indicate NUTS-1 regions that have not been included in the forecasting because of insufficient impact reports. For the acronym of NUTS-1 regions in Germany, see supplementary Fig. 1.

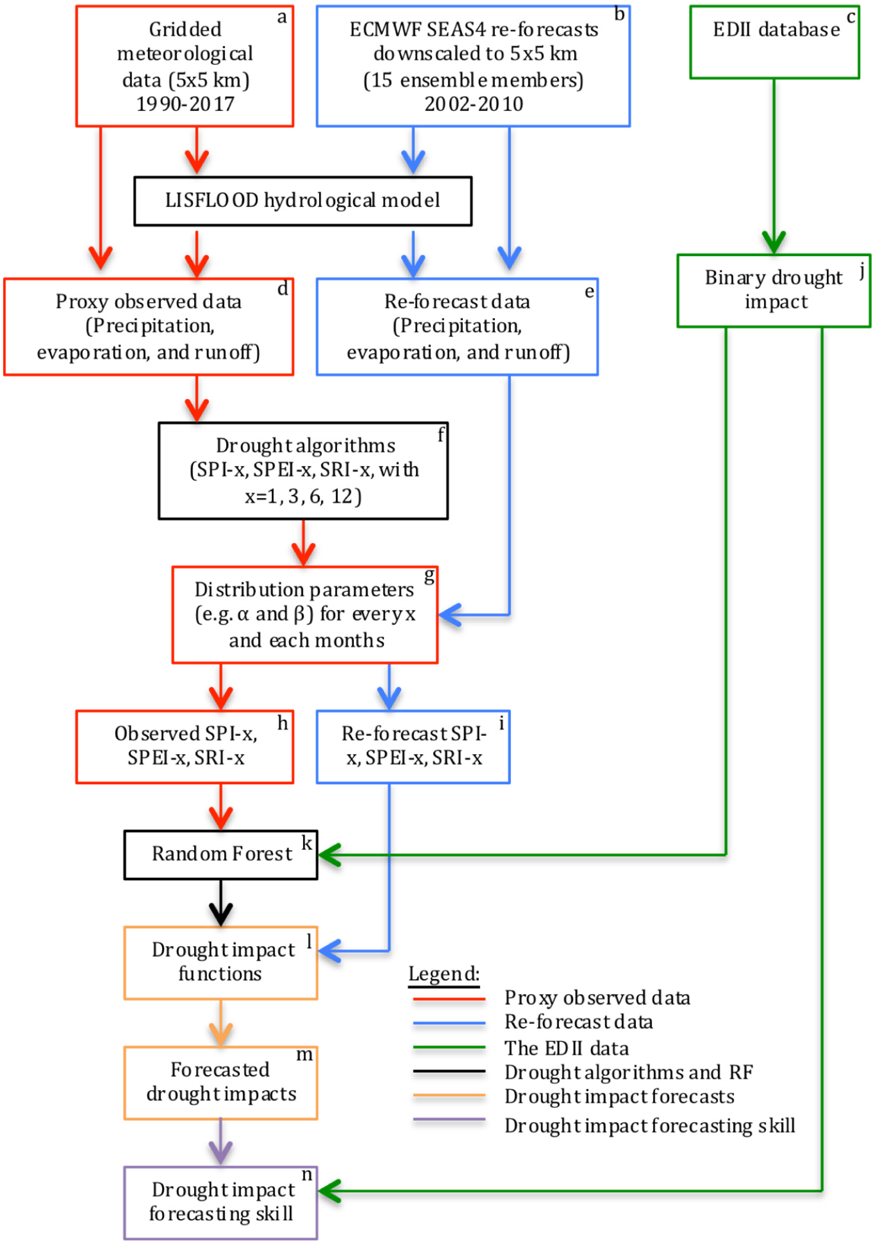

Supplementary Figure 5. Flowchart showing data and methods.

Supplementary Notes

Analyses in this study were carried out based on the German NUTS-1 regions. The NUTS regions are geo-coded standard regions developed by the European Union[2]. In Germany, the NUTS-1 regions correspond with the federal states (Supplementary Fig. 1). Figure 1 shows the German NUTS-1 regions and their acronyms.

Collection of drought impact reports and their entries in the EDII[1] is still an on-going process. The text-based reports are obtained from various sources, such as governmental reports, NGO reports, newspapers, digital media, and scientific papers. Impact entries in the EDII must contain: (1) temporal reference that can be indicated by month, season, or year; and (2) spatial reference, which is the location of the reported impacts. This can be either referred to different levels of geographical regions using the European Union NUTS standard (Nomenclature of Units for Territorial Statistics) or specified by rivers and lakes. The drought impact reports are divided into 15 impact categories: (1) agriculture and livestock farming, (2) forestry, (3) freshwater aquaculture and fisheries, (4) energy and industry, (5) waterborne transportation, (6) tourism and recreation, (7) public water supply, (8) water quality, (9) freshwater ecosystems, (10) terrestrial ecosystem, (11) soil system, (12) wildfires, (13) air quality, (14) human health and public safety, and (15) conflicts (Supplementary Fig. 2). Supplementary Figure 2 shows that in Germany most drought impacts occurred during the summer period and mainly in the southern and western regions (BV, BW, and BB, see Supplementary Fig. 1 for the acronyms). The most frequent reported drought impacts are in the categories agriculture and livestock farming, freshwater ecosystems, public water supply, and water-borne transportation.

Availability of reported impact information in the EDII appears to vary across Europe. This results in temporal and spatial biases within the EDII. For the present study, Germany was selected to explore the potential of drought impact forecasting, as it is one of the most documented countries within the EDII.

An example of time series of drought events in the German NUTS-1 regions RP and BB is presented in Supplementary Figure 3. These events were calculated using proxy observed data for Standardized Precipitation Index and Standardized Runoff Index accumulated over 6 months (SPI-6 and SRI-6, respectively). The optimal accumulation period for standardized drought indices depends on catchment characteristics (fast versus slowly-responding catchments), but also on the impacted sector. For some sectors, which largely depend on soil moisture, an accumulation period of 3 months (SPI-3) fits well, for other sectors that are more influenced by groundwater, or groundwater-fed rivers, longer accumulation periods are selected (e.g. SPI-6). For instance, the heat maps compiled by Ref. 3 show that accumulation periods over 6 months (SPI-x, x>6) are typical for groundwater. The temporal evolution of the SPI-6 and SRI-6 shows that droughts occurred in RP and BB in 2003, 2006, and 2008.

Drought impact forecasting functions have been developed for the German NUTS-1 regions with sufficient impact data for the drought impact groups: (1) agriculture and livestock farming, and forestry; (2) energy and industry, water-borne transportation, and public water supply; (3) water quality, freshwater ecosystem, and terrestrial ecosystem; and (4) wildfire, air quality, and human health and public safety. These functions were trained using observed drought events derived from drought indices (SPI-x, SPEI-x, and SRI-x, with x=1, 3, 6, 12), years in drought, and months in drought from 1990 to 2017 as predictors, and binary drought impact time series. Supplementary Figure 4 illustrates the predictor importance for every region and for selected merged impact categories (impact groups), which are generated with the Random Forest algorithm. The dark gray boxes indicate that insufficient data are available for a certain impact group and NUTS-1 region to develop drought impact forecasting functions.

Overall, the year of impact occurrence seems to be a good predictor for all the impact groups (most right bar in each histogram). This is the result of temporal bias within the EDII. For impact Group 1, accumulation periods of 3 and 6 months are best linked with the impact (Supplementary Fig. 4a). SPEI and SRI are better drought impact predictors than the SPI. The accumulation period of 6 months appears to be the best predictor for impact Group 2, closely followed by the accumulation periods of 3 and 12 months (Supplementary Fig. 4b). Drought indices SPEI and SRI are best linked to this impact group. As this category is well covered across Germany, spatial trends can be found. For the more southern regions, accumulation periods of 3 and 6 months are best linked. The northern regions are well correlated with longer accumulation periods (6 and 12 months). Supplementary Figure 4c shows that best predictors for impact Group 3 have accumulation periods of 3 and 6 months. Like the other two impact groups, the drought indices SPEI and SRI have better predictive power than SPI. No clear spatial trends can be found across regions for this impact group. For impacts related to fires, health, and air quality (Group 4), predictors with short accumulation period are most important for forecasting this drought impact, closely followed by predictors with accumulation periods of 6 months (Supplementary Fig. 4d).

In our study, we used methods that are already well established. As described in the Method section, data and methods used in this study are similar with studies described in Ref. 4, 5, 6, and 7. Supplementary Figure 5 shows the flowchart describing the data and methods.

Supplementary References

1. Stahl, K. *et al*. Impacts of european drought events: insights from an international database of text-based reports. *Nat. Hazards Earth Syst. Sci.* **16**, 801–819, DOI: https://doi.org/10.5194/nhess-16-801-2016 (2016).
2. Eurostat. Regions in the european union: Nomenclature of territorial units for statistics nuts 2010/eu-27. *Eur. Union* (2011).
3. Bloomfield, J. P. & Marchant, B. P. Analysis of groundwater drought building on the standardised precipitation index approach. *Hydrol. Earth Syst. Sci.* **17 (12),** 4769-4787 (2013).
4. Arnal, L. *et al*. Skilful seasonal forecasts of streamflow over europe? *Hydrol. Earth Syst. Sci.* **22**, 2057–2072, DOI: https://doi.org/10.5194/hess-22-2057-2018 (2018).
5. Bachmair, S. *et al*. Drought indicators revisited: the need for a wider consideration of environment and society. *WIREs Water* **3**, 516–536, DOI: https://doi.org/10.1002/wat2.1154 (2016).
6. Bachmair, S., Svensson, C., Prosdocimi, I., Hannaford, J. & Stahl, K. Developing drought impact functions for drought risk management. *Nat. Hazards Earth Syst. Sci.* **17**, 1947–1960, DOI: https://doi.org/10.5194/nhess-17-1947-2017 (2017).
7. Van Hateren, T., Sutanto, S. J. & Van Lanen, H. A. J. Evaluating uncertainty and robustness of seasonal meteorological and hydrological drought forecasts at the catchment scale-case catalonia (spain). *Env. Int.* (accepted).
